# Secondary signs on preoperative CT as predictive factors for febrile urinary tract infection after ureteroscopic lithotripsy

**DOI:** 10.1186/s12894-020-00701-7

**Published:** 2020-08-27

**Authors:** Jin Woo Kim, You Jin Lee, Yun-Sok Ha, Jun Nyung Lee, Hyun Tae Kim, So Young Chun, Bum Soo Kim

**Affiliations:** 1 Department of Urology, Kyungpook National University Hospital, 130 Dongdeok-ro, Jung-gu, Daegu, 41944 South Korea; 2 Department of Urology, Pohang Semyeng Christianity Hospital, Pohang, South Korea; 3 Department of Urology, School of Medicine, Kyungpook National University, Daegu, South Korea; 4 Department of Urology, Kyungpook National University Chilgok Hospital, Daegu, South Korea; 5 BioMedical Research Institute, Joint Institute for Regenerative Medicine, Kyungpook National University Hospital, Daegu, South Korea

**Keywords:** Urolithiasis, Ureteroscopy, Urinary tract infections, Computed tomography (CT)

## Abstract

**Background:**

Febrile urinary tract infection (UTI) is one of the most common complications after ureteroscopic lithotripsy (URS). We evaluated the effect of secondary signs on preoperative computed tomography (CT) for febrile UTI after URS.

**Methods:**

In total, 182 patients who underwent URS for ureteral stones from January 2013 to December 2015 were retrospectively included in this study. These patients were divided into two groups according to the presence of postoperative febrile UTI after URS. We compared the clinical factors, stone factors, and secondary signs between the groups. Predictive factors for febrile UTI after URS were analyzed using a multivariate logistic regression model.

**Results:**

Febrile UTI occurred in 26 of the 182 patients. In univariate analysis, presence of comorbid chronic kidney disease (CKD) and stone size were significantly different between UTI and non-UTI groups. Among secondary signs, presence of hydroureter, perinephric fat stranding, periureteral fat stranding, and tissue rim sign were significantly different between the groups. In multivariate logistic regression analysis, comorbid CKD, stone size, perinephric fat stranding, and tissue rim sign were independent predictive factors for febrile UTI after URS.

**Conclusion:**

This study demonstrated that secondary signs including perinephric fat stranding and tissue rim sign on preoperative CT, CKD, and stone size are independent predictive factors for febrile UTI after URS.

## Background

Ureteroscopic lithotripsy (URS) is an effective and safe minimally invasive modality for the management of ureter stones. The first ureteroscopic procedure was introduced in the 1960s, and it has been currently considered as the preferred treatment modality for the management of ureter stones. However, various complications can occur after URS, of which febrile urinary tract infection (UTI) is the most common complication, which can worsen with sepsis in serious cases [1].

Unenhanced helical computed tomography (UHCT) is one of the most useful imaging modalities for the diagnosis of urinary stones. UHCT provides information regarding urinary stones, including their location, sizes, number, and attenuation values, with high sensitivity (95–98%) and specificity (96–100%), as shown by previous studies [2, 3]. Since the 1990s, with the development of image processing and analysis of UHCT, several studies have reported the analysis of secondary signs of ureteral obstruction on UHCT for urinary stones as result of physiologic changes in the obstructed kidney [4–6]. In addition, various studies have suggested the clinical influence of secondary signs or the correlation between stone factors and secondary signs on UHCT for urinary stone [7–10]. However, to date, the impact of secondary signs on postoperative febrile UTI after URS for ureter stones has not been evaluated.

We hypothesized that compared to the obstructed kidney without secondary signs on preoperative UHCT, that which represents secondary signs on the image can more easily cause febrile UTI after URS. In this study, we evaluated the effect of secondary signs on preoperative UHCT on febrile UTI after URS based on the experience of our center, and analyzed the possible predictive factors for febrile UTI after URS, including the secondary signs on preoperative CT.

## Methods

The Institutional Review Board of the Kyungpook National University Hospital approved the study protocol based on the Declaration of Helsinki (approval number: KNUH 2019–05-001). In total, 182 patients who underwent URS for ureteral stones in our center from January 2013 to December 2015 were retrospectively included in this study. Patients who underwent retrograde intrarenal surgery due to renal stones were excluded. Patients with preoperative ureteral stent or percutaneous nephrostomy were also excluded in this study. The definition of febrile UTI in this study was occurrence of high fever (> 38 °C) with pyuria within 1 week after URS without other infectious signs except UTI. A single surgeon performed all operations using an 8.5-Fr semi-rigid ureteroscope (Karl Storz, Tuttlingen, Germany) with 200-μm holmium laser (Lumenis, Tel Aviv, Israel). We divided these patients into two groups according to the presence of postoperative febrile UTI within 2 weeks after URS; Group A (*n* = 26) included patients with febrile UTI after URS and Group B (*n* = 156) included patients without febrile UTI after URS.

**Fig. 1:**
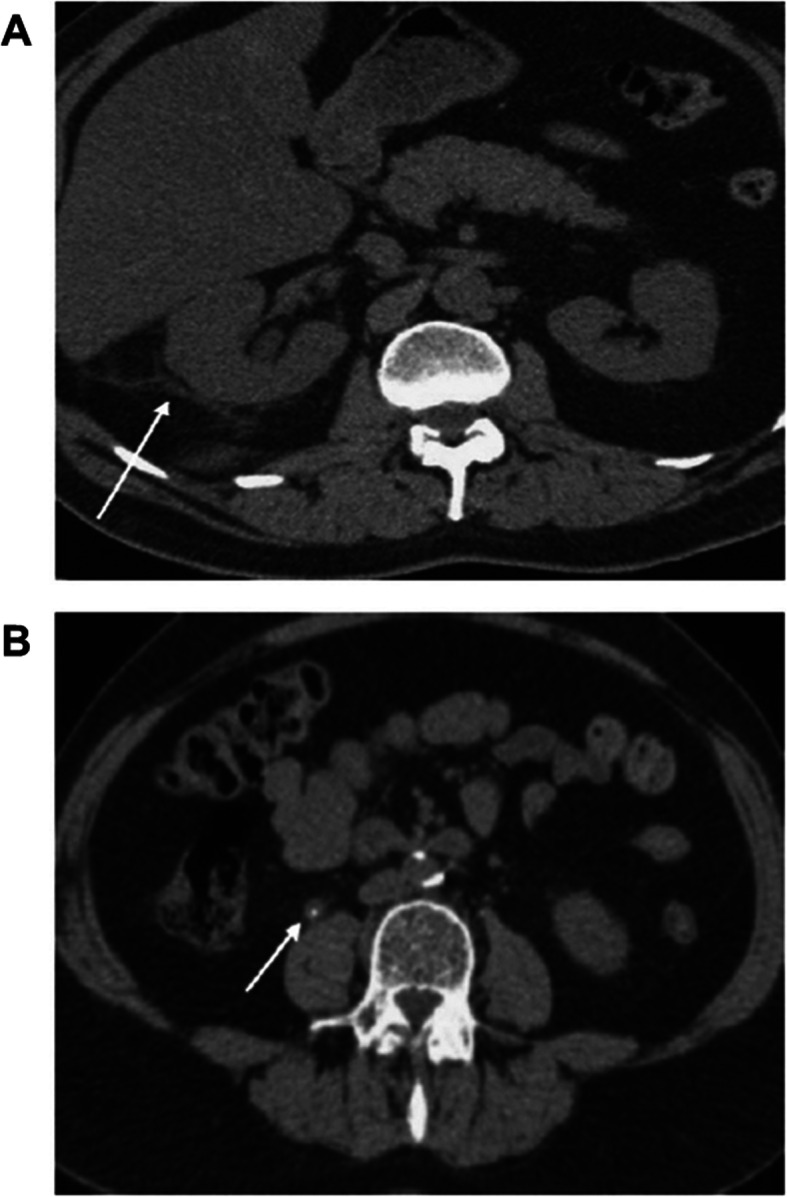
Features of perinephric fat stranding (**a**), and tissue rim sign (**b**) secondary to ureteral stone. The white arrows are pointing the perinephric fat stranding (**a**) and tissue rim sign (**b**)

We evaluated and compared the preoperative clinical data and stone characteristics between patients in the two groups through a review of medical records. The clinical data included age; gender; body mass index (BMI); comorbidities such as hypertension (HTN), diabetes (DM), chronic kidney disease (CKD); and history of previous acute pyelonephritis (APN) and stone surgery; the stone characteristics included laterality, location, mean number of stones, size, and Hounsfield units (HU). Secondary signs included hydronephrosis, hydroureter, unilateral enlargement, perinephric fat stranding, periureteral fat stranding, and tissue rim sign (Fig. 1).

All abdominal UHCT examinations were performed preoperatively with 5-mm cut slices for axial images and 3-mm cut slices for coronal images. The stone size was determined by measuring the longest axis, and HU was evaluated on axial image in the mid-lateral aspect of each kidney using the maximal region on preoperative CT image. Perinephric and periureteric fat stranding were defined as linear areas of soft tissue attenuation in the perinephric and periureteric space, respectively [6]. Positive tissue rim sign was defined as annular soft tissue attenuation (20–40 HU) caused by an edematous ureteral wall surrounding the stone [6]. All the interpretations of preoperative UHCT were simultaneously performed and discussed by one radiologist and one urologist, and presence of secondary signs was determined by the consensus of a radiologist and urologist.

Before URS, all patients were evaluated through physical examination, routine blood tests, urinalysis, urine culture, and radiologic images, including simple X-ray (KUB), and UHCT. Patients whose urine cultures demonstrated bacterial growth on preoperative evaluation were treated with appropriate antibiotics, and the surgery was performed after sterile urine was confirmed. Fluoroquinolone was routinely used as a prophylactic antibiotic for all patients on the day of the surgery.

The surgery was performed under general or spinal anesthesia in the lithotomy position for all patients. After cystoscopy, the hydrophilic guidewire was inserted into the ureter. A semi-rigid ureteroscope was introduced to visualize the ureter and facilitate its placement. Lithotripsy was performed using a laser lithotripter. Irrigation during surgery was manually provided by a surgical assistant (urologic resident) using 50-ml syringe. The assistant was previously trained to maintain the irrigation pressure between 60 and 120 cmH_2_O depending on the surgical situation, such as visual clearness and possibility of stone retropulsion. A 1.9-F nitinol stone basket (Zero-tip; Boston Scientific, Spencer, IN, USA) was used to remove remnant stone fragments from the urinary tract. At the end of the surgery, a 6-F double-J stent was routinely placed and maintained for 1 or 2 weeks in all patients.

Data were evaluated using SPSS 18.0 (SPSS, Inc., Chicago, IL, USA). Chi*-*square test was used to determine the difference in proportions for categorical data, while continuous variables were assessed using Wilcoxon test. Multivariate logistic regression analysis using forward selection was performed to identify the risk factors of febrile UTI after URS. A *P* value < 0.05 (two-tailed) was considered statistically significant.

## Results

Febrile UTI occurred in 26 of the 182 patients. The patient characteristics, stone characteristics, and presence of secondary signs were compared between UTI and non-UTI groups, and are presented in Table 1. Presence of comorbid CKD was 6/26 (23.1%) and 11/156 (7.1%) in group A and B, respectively (*p* = 0.020). Mean stone size was 13.19 ± 8.95 mm and 9.41 ± 3.80 mm in group A and B, respectively (*p* < 0.001) Both prevalence of comorbid CKD and mean stone size were significantly different between groups A and B. Among secondary signs, hydroureter was found in 25/26 (96.2%) and 92/156 (58.3%) in group A and B, respectively (*p* = 0.006). Perinephric fat stranding was observed in 23/26 (88.5%) and 69/156 (44.2%) in group A and B, respectively (*p* < 0.001). Periureteral fat stranding was seen in 22/26 (84.6%) and 91/156 (58.3%) (*p* = 0.015), and tissue rim sign was observed in 23/26 (88.5%) and 98/156 (62.8%), respectively (*p* = 0.010). All of these secondary signs were significantly different between groups A and B. There were no significant differences in the stone-free rate (96.2% vs. 84.9%) and operation time (54.26 min vs. 59.00 min) between groups A and B.

**Table 1:** Comparison of preoperative patient characteristics and secondary signs between groups A and B

Characteristics	Group A (*n* = 26)	Group B (*n* = 156)	*P*-value
Age (yr)	58.58 ± 14.29	57.44 ± 15.26	0.722
Gender (Male/Female)	17/9	102/54	1.000
BMI (kg/m^2^)	25.43 ± 4.12	24.83 ± 3.72	0.453
HTN	15 (57.7%)	61 (39.1%)	0.088
DM	33 (21.2%)	6 (23.1%)	0.792
CKD	6 (23.1%)	11 (7.1%)	0.020
Previous APN	10 (38.5%)	34 (21.8%)	0.066
Previous stone operation	4 (15.4%)	16 (10.3%)	0.439
Hounsfield unit	1011.08 ± 400.82	1033.44 ± 423.38	0.963
Stone laterality (%)			
Right	10 (38.5%)	80 (51.3%)	
Left	12 (46.2%)	69 (44.2%)	
Both	4 (15.3%)	7 (4.5%)	
Stone position (%)			
Upper ureter	16 (61.5%)	71 (45.5%)	
Mid ureter	2 (7.7%)	24 (15.4%)	
Lower ureter	8 (30.85)	61 (39.1%)	
Mean number of stones	1.34 ± 0.45	1.38 ± 0.39	0.823
Size (mm)	13.19 ± 8.95	9.41 ± 3.80	0.000
Hydronephrosis	25 (96.2%)	126 (80.8%)	0.053
Hydroureter	25 (96.2%)	92 (58.3%)	0.006
Unilateral enlargement	6 (23.1%)	20 (12.8%)	0.166
Perinephric fat stranding	23 (88.5%)	69 (44.2%)	< 0.001
Periureteral fat stranding	22 (84.6%)	91 (58.3%)	0.015
Tissue rim sign	23 (88.5%)	98 (62.8%)	0.010

*BMI* Body mass index, *HTN* Hypertension, *DM* Diabetes mellitus, *CKD* Chronic kidney disease, *APN* Acute pyelonephritis

In the multivariate logistic regression analysis, comorbid CKD (OR = 3.739, 95%CI = 1.030–13.572), stone size (OR = 1.101, 95%CI = 1.009–1.201), perinephric fat stranding (OR = 7.622, 95%CI =2.104–27.605), and tissue rim sign (OR = 5.003, 95%CI = 1.289–19.413) were found to be independent predictive factors for febrile UTI after URS (Table 2).

**Table 2:** Multivariate logistic regression analysis for risk factors of febrile urinary tract infection after ureteroscopic lithotripsy

Characteristics	HR (95% CI)	*P*-value
CKD	3.739 (1.030–13.572)	0.045
Size (mm)	1.101 (1.009–1.201)	0.031
Perinephric fat stranding	7.622 (2.104–27.605)	0.002
Tissue rim sign	5.003 (1.289–19.413)	0.020

*HR* Hazard ratio, *CI* Confidence interval, *CKD* Chronic kidney disease

## Discussion

Postoperative febrile UTI after URS is one of the most frequent and important complications to be considered [11, 12], and many studies have researched the risk factors associated with postoperative febrile UTI after URS. Bloom et al. reported that the most common complication after URS for readmission was fever and pain, accounting for 43.8% [13]. Although no study has specifically reported the correlation of secondary signs on preoperative UHCT with febrile UTI after URS, various related studies have been reported. Recently, several studies have demonstrated that secondary sign-associated urinary stone is the result of the obstructed kidney, and that it can provide data on the degree of the ureteral obstruction [14–16]. Eugene et al. reported that the secondary signs on UHCT were associated with concurrent ureteral lesions such as severe mucosal edema, strictures, ureteral polyps, or submucosal stones [7]. Based on the results of these studies, we presumed that there could be a correlation between secondary signs and postoperative febrile UTI.

Among the clinical factors in our study, CKD showed a significant difference when febrile and non-febrile UTI groups were compared. CKD is a state of reduced tubular clearance, with decline in renal function. Although the exact mechanism of and relationship between CKD and post-operative UTI have not been well-investigated, we presume that deteriorated renal function and reduced tubular clearance after URS may delay the washout of irrigation fluid and stone fragments, which can be a source of infection, and may increase the risk of postoperative UTI. The stone size was another predictor of postoperative UTI in our study. Irrigation during URS increases renal pelvic pressure, potentially causing intrarenal, pyelovenous, and pyelolymphatic backflow. The amount of irrigation during URS can increase as the stone size increases, even the pressure of irrigation also can increase since manual irrigation was performed in this study; therefore, larger stone burden requires longer operation time and massive irrigation during the procedure, increasing the absorption of infected urine.

Of all secondary signs, perinephric fat stranding and tissue rim sign were found to be predictive factors for febrile UTI after URS. Perinephric fat stranding, observed in 36–82% of adult patients with ureter stone [3, 17–21], was defined as linear areas of soft tissue attenuation in the perinephric space and increased density in the surrounding perirenal adipose tissue. The changes in the perinephric space are caused by the fluid released within the bridging septa of the perinephric fat as a result of increased lymphatic pressure, inflammation, and edema in the ureteral wall surrounding the stones. A 34–76% incidence of tissue rim sign, defined as about 2-mm rim of soft tissue attenuation (20–40 HU), has been reported in cases of ureter stone [22], and this sign is a useful indicator to distinguish ureter stone from phleboliths. Tissue rim sign is known to be the result of inflammatory and edematous changes in the ureteric wall, caused by contact with the obstructing ureteral stone. Consequently, the perinephric fat stranding and tissue rim sign on preoperative UHCT reflect the inflammatory changes resulting from the impacted stone of the urinary tract. Therefore, the presence of these two signs on preoperative CT indicates febrile UTI after URS.

This study has several limitations. First, the relatively small number of patients, especially in the febrile UTI group, limited the statistical significance of some findings. Second, the results of struvite stone analysis were not compared between the groups owing to lack of data. Instead, we indirectly compared preoperative stone characteristics using HU measured by preoperative CT. Finally, as this was a retrospective study, we did not perform a randomized case-controlled study with a detailed analysis for ureteroscopic findings such as impacted stone. Moreover, since the interpretations of secondary signs were not performed preoperatively, the CT images were reevaluated retrospectively. Although the interpreters did not know the presence of postoperative UTI, while they reevaluated CT images, it could be a potential bias. However, to the best of our knowledge, this is the first study to analyze the relationship between secondary signs and postoperative febrile UTI. We believe that the results of this study can suggest potential risk factors of postoperative UTI after URS, which may help reduce the postoperative complications.

## Conclusions

This study demonstrated that secondary signs including perinephric fat stranding and tissue rim sign on preoperative CT, CKD, and stone size are independent predictive factors for febrile UTI after URS for ureter stone. UHCT is a useful diagnostic modality for ureteral stone, and the measurement of secondary signs on preoperative UHCT could help predict febrile UTI after URS.

## Data Availability

The datasets used and analyzed in this study are available from the corresponding author on reasonable request.

## Abbreviations

URS: Ureteroscopic lithotripsy
UTI: Urinary tract infection
UHCT: Unenhanced helical computed tomography
BMI: Body mass index
HTN: Hypertension
DM: Diabetes mellitus
CKD: Chronic kidney disease
APN: Acute pyelonephritis
HU: Hounsfield units
HR: Hazard ratio
CI: Confidence interval
